# Episodic itch in a case of spinal glioma

**DOI:** 10.1186/1471-2377-13-124

**Published:** 2013-09-23

**Authors:** Stefan Wolking, Holger Lerche, Marcel Dihné

**Affiliations:** 1 Department of Neurology and Epileptology, Hertie-Institute for Clinical Brain Research, University of Tuebingen, Hoppe-Seyler-Str.3, Tuebingen 72076, Germany

**Keywords:** Itch, Spine, Glioma, MRI, Gabapentin

## Abstract

**Background:**

Itch is a frequent complaint reported by patients and is usually ascribed to dermatological or metabolic causes. In neurological disorders, however, it is a very unusual symptom and thus its neurological aetiology is likely to be overlooked. There are only very few reports about permanent itch related to lesions of the central nervous system. To our knowledge we report the first case of episodic itch associated with a central nervous lesion.

**Case presentation:**

A 74-year-old female suffered from long-standing episodes of itch of the dermatomes C2 to C6 on the right side that was refractory to any treatment. On occurrence it propagated in a proximal to distal fashion. Between the episodes the patient was asymptomatic. MRI of the cervical spine uncovered a spinal glioma that matched the location of the symptoms. Treatment with gabapentin led to a prompt reduction of the symptoms.

**Conclusion:**

Patients with intractable pruritus and dermatomal presentation ought to undergo neurological examination and spinal cord imaging. Thus, ongoing frustrating and sometimes even harmful treatment trials could be avoided.

## Background

Chronic itch is a common symptom and often proves to be a diagnostic and therapeutic challenge. It is often related to dermatologic aetiologies. In the absence of skin alteration, metabolic or endocrinologic diseases are frequently diagnosed.

In neurological disorders, however, itch is a rather unusual symptom within which peripheral neural affections such as postherpetic itch after shingles [1] or radiculopathy due to osteoarthritis [2] account for the major part. In contrast, central itch is even far less frequent and, predominantly caused by cerebral lesions. It is often caused by cerebral lesions such as brain stem infarction [3], multiple sclerosis [4], brainstem glioma [5] or supratentorial cerebral abscess [6].

Only few cases are reported on pruritus associated with spine tumours, such as intramedullary astrocytoma in children [7] and intramedullary cervical ependymoma in adults [8]. Other spinal lesions may be associated with pruritus as well, such as cervical cavernoma [9], cavernous hemangioma [10] and vascular malformation [11].

In all the reported cases, permanent and not episodic itching – as observed in our patient – has been reported. It is also unusual that no other sensory symptoms occurred between the episodes.

## Case presentation

A 74-year-old female was admitted to our department with intractable pruritus of the right arm, ear lobe and the right occiput. For 5 to 6 years the pruritus had appeared episodically about every two days lasting for several hours. It usually emerged from the right shoulder and then propagated along the radial side of the arm down to the wrist within few minutes and persist for several hours. Occasionally, the itch emanated from further above, originating from the right side of the back of the head including the right ear lobe with propagation down to the right arm as described before. Apart from scratch marks no rash or skin alterations had been observed in the affected region. Between the episodes, the patient was asymptomatic.

The patient had a history of multiple dermatologic treatment approaches. Amongst others, allergic contact dermatitis and food intolerance syndrome as well as mastocytosis had been suspected, but could not be confirmed. Treatment with topic and oral steroids had shown no positive effect. She reported that intake of the histamine antagonist *desloratadine* at the onset of symptoms would lead to a relief after two to three hours. Nonetheless, without drug intake, the symptoms would be self-limiting as well. Thus, there was no evidence of any benefit deriving from antihistaminic treatment.

Examination revealed slightly elevated tendon reflexes and Hoffmann’s reflex of the right upper limb and a minor clumsiness of the right hand. Sensibility was intact. In the arm extension test, a heaviness of the right arm was reported, however there was no objectifiable motor deficit. Lower Limb examination showed no reflex abnormalities, no motor deficit and negative pyramid signs. Skin examination revealed no abnormalities.

**Figure 1:**
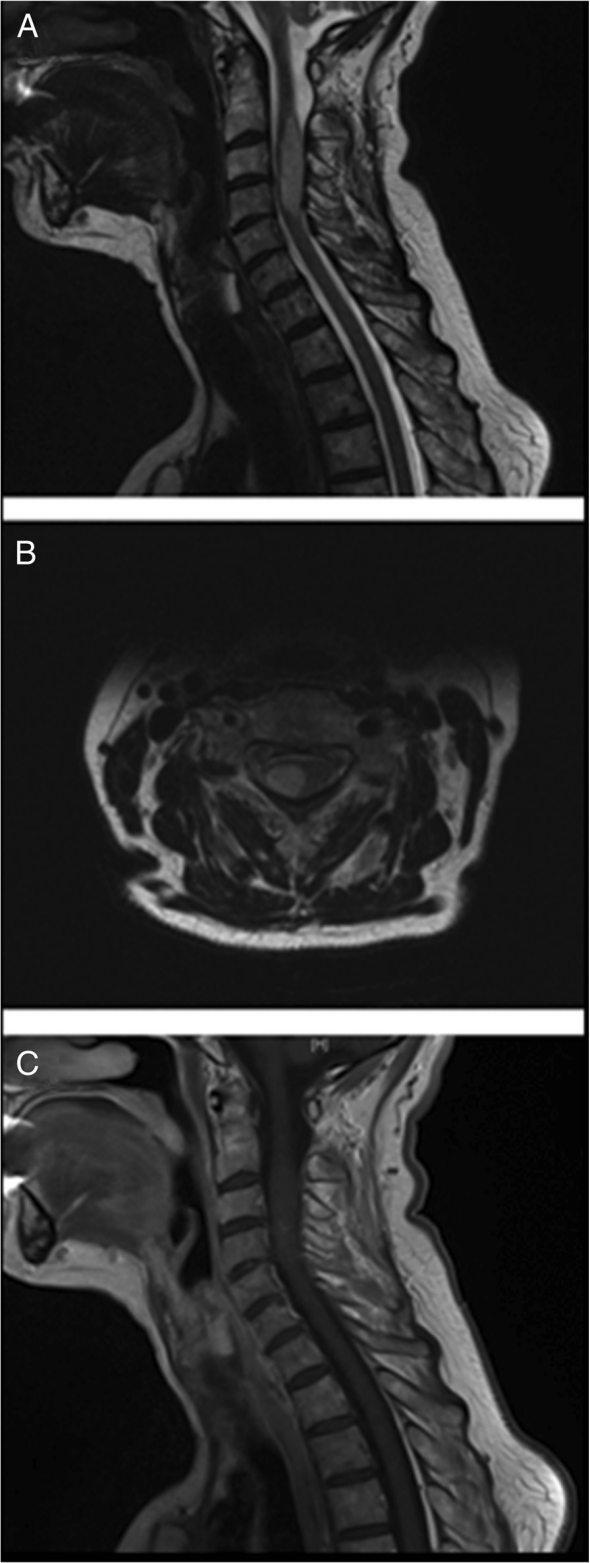
**MRI of cervical spine: Sagittal and axial T2-weighted MRI images show an intramedullary tumor (A and B).** T1-weighted images with contrast enhancement show a slight contrast uptake **(C)**.

Cervical MRI revealed a right-sided T2-hyperintense intramedullar tumour mass extending from C2 to C5 with an axial diameter of 7–8 mm, which showed a slight contrast enhancement (Figure 1). There were no signs of bleeding. Morphologically, the mass had the aspect of a spinal glioma. Cerebral MRI was normal.

Somatosensory evoked potentials (SSEP) of the right median nerve showed an increased latency for C7 and C2 whereas cortical recordings were normal. Tibial nerve SSEP showed a symmetrical increase of latency on cortical recordings. Motor evoked potentials (MEP) revealed a borderline decrease of amplitude to the right arm. In order to rule out an inflammatory etiology CSF analysis was perfornerd. It showed no signs of inflammation, atypical cells or prior bleeding.

The patient refused any surgical treatment or biopsy. The long persistence of the symptoms suggested a low growth-rate of the tumour. A control MRI was performed after four months without any change of the tumour mass.

We started the patient on gabapentin (1200 mg/d) which provoked initially a rapid reduction of the pruritus. After 3 months, however, episodic pruritus recurred and gabapentin was discontinued due to fatigue. Afterwards, pregabalin was introduced but withdrawn shortly due to side effects. Oxcarbazepine is now intended as the next drug.

## Discussion

We report a case of longstanding episodic pruritus in association with a cervical intramedullary glioma. To our knowledge it is the first case to report of episodic – not permanent – itch due to a lesion of the central nervous system.

The propagation of the itch within a few minutes in a proximal-distal direction is reminiscent of focal seizures (somato-sensory auras) with spreading paraesthesias, as a sensory Jacksonian march.

Since the cervical lesion between the vertebrae C2 to C5 coincides with the clinical presentation of sensory symptoms restricted to dermatomes C2 to C6, although C6 dermatome was not completely affected leaving out the right thumb, we hypothesize that electrical phenomena similar to epileptic seizures or spreading depression in the cortex might cause the episodic symptoms. The excellent initial response to gabapentin supports this hypothesis. However, gabapentin is used to treat peripheral neuropathy as well and thus its efficacy in this particular case does not prove a central origin of the itch.

Alternatively, variations of the local intraspinal pressure might cause the observed fluctuation.

The existence of spinal cord seizures in humans has been postulated by several authors in association with multiple sclerosis [12], spinal ischemia [13] and traumatic injury [14]. In most cases the seizures were focal-motor or myoclonic and were often associated with dysaesthesia in the affected limb [12]. In several cases a response to antiepileptic drugs such as carbamazepine and benzodiazepines was reported [12,14].

## Conclusions

Chronic itch is a very trying and cumbersome burden for the affected patients and a diagnostic and therapeutic challenge for the practitioners. If dermatological and metabolic tests yield no explanation diagnostics should be extended. Especially, patients with episodic occurrence of itch, a dermatomal localization and progression should be referred to neurological examination and eventually spinal cord imaging. These patients may profit from antiepileptic treatment.

## Consent

Written informed consent was obtained from the patient for publication of this Case report and the accompanying images. A copy of the written consent is available for review by the Editor of this journal.

## Competing interests

The authors declare that they have no competing interest.

## Authors’ contributions

SW drafted the manuscript, collected the data and performed the literature search. HL revised the paper. MD revised the paper. All authors read and approved the final manuscript.

## Pre-publication history

The pre-publication history for this paper can be accessed here:

http://www.biomedcentral.com/1471-2377/13/124/prepub
